# Cadmium induces neuronal cell death through reactive oxygen species activated by GADD153

**DOI:** 10.1186/1471-2121-14-4

**Published:** 2013-01-22

**Authors:** Seungwoo Kim, Hyo-Soon Cheon, So-Young Kim, Yong-Sung Juhnn, Young-Youl Kim

**Affiliations:** 1 Center for Biomedical Science, Division of Brain Diseases, National Institute of Health in Korea (KNIH), Osong Health Technology Administration Complex, 187 songsaengmyeong2(i)-ro, Gangoe-myeon, Cheongwon-gun, Chungcheongbuk-do, 363-951, Korea; 2 Department of Biochemistry and Molecular Biology, Cancer Research Institute, Seoul National University College of Medicine, Seoul, 110-799, Korea

**Keywords:** Cadmium, ROS, ER, Bak

## Abstract

**Background:**

Cadmium(Cd), a heavy metal, which has a potent harmful effects, is a highly stress-inducible material that is robustly expressed following disruption of homeostasis in the endoplasmic reticulum (ER) (so-called ER stress). The mechanism Cd induced cell death of neuroblastoma cells complex, involving cellular signaling pathways as yet incompletely defined but, in part, involving the generation of reactive oxygen species (ROS). Several studies have correlated GADD153 expression with cell death, but a mechanistic link between GADD153 and apoptosis has never been demonstrated.

**Results:**

SH-SY5Y cells were treated Cd led to increase in intracellular ROS levels. ROS generation is not consistent with intracellular [Ca^2+^]. The exposure of neuroblastoma cells to Cd led to increase in intracellular GADD153 and Bak levels in a doses and time dependent manner. The induction of these genes by Cd was attenuated by NAC. Cd-induced apoptosis is decreased in GADD153 knockdown cells compared with normal cells. The effect of GADD153 on the binding of C/EBP to the Bak promoters were analyzed ChIP assay. Basal constitutive GADD153 recruitment to the –3,398/–3,380 region of the Bak promoter is observed in SH-SY5Y cells.

**Conclusions:**

The exposure of SH-SY5Y cells to Cd led to increase in intracellular ROS levels in a doses and time dependent manner. The generation of ROS result in the induction of GADD153 is causative of cadmium-induced apoptosis. GADD153 regulates Bak expression by its binding to promoter region (between −3,398 and −3,380). Therefore, we conclude that GADD153 sensitizes cells to ROS through mechanisms that involve up-regulation of BAK and enhanced oxidant injury.

## Background

ROS are constantly generated under normal conditions as a consequence of aerobic metabolism, and the most common ROS types are superoxide anions (O2–), hydrogen peroxide (H_2_O_2_) and hydroxyl radicals (HO–) [1]. ROS can react with DNA, proteins, carbohydrates, and lipids in a destructive manner due to their high levels of chemical reactivity. ROS participate in the modulation of apoptosis following treatment with various agents including Fas, ultraviolet, and chemotherapeutic drugs [2]. Several previous studies also indicated involvement of ROS in cadmium-induced renal tubular injury. For example, exposure of LLC-PK1 cells to cadmium caused generation of ROS [3], which was associated with a decrease in glutathione levels and consequent cellular death [4]. Another report showed that Cd-triggered apoptosis of tubular cells is inhibited by an antioxidant [5].

However, currently, it is unknown whether and how oxidative stress is linked to ER stress and, if so, what kind of ROS are involved in the induction of apoptosis in cadmium-exposed cells. GADD153 is a member of the C/EBP family of bZIP transcription factors, and its expression is induced to high levels by ER stress [6]. GADD153 forms hetero-dimers with other C/EBP-family transcription factors via bZIP-domain interactions, which suppresses their binding to C/EBP sites in DNA, while promoting binding to alternative DNA sequences for target gene activation [7]. Consequently, GADD153 inhibits expression of genes responsive to C/EBP-family transcription factors, while enhancing expression of other genes containing the consensus motif 5′-(A/G)(A/G)(A/G)TGCAAT(A/C)CCC-3′. One relevant target may be *bcl2*, whose expression is suppressed by GADD153, at least in some cellular contexts [8]. While capable of inducing apoptosis and contributing to cell death in several scenarios involving ER stress, GADD153 is not essential for cell death induced by ER stress, as demonstrated by the observation that *perk*^−/−^ and eIF2α(S51A) knock-in cells are hypersensitive to ER stress–induced apoptosis but fail to induce gadd153 gene expression [9,10]. Splenic lymphocytes from GADD153^−/−^ mice are relatively resistant to LPS-induced apoptosis, which is associated with increased Bax and Bak mRNAs and decreased Bcl-2 mRNA (S Oyadomari *et al*., unpublished observation). Furthermore, the activation of GADD153 leads to translocation of Bax protein from the cytosol to the mitochondria [8]. Thus, GADD153-mediated death signal is finally transmitted to the mitochondria, which functions as an integrator and amplifier of the death pathway.

Clearly, there is great need for more definitive evidence of the involvement of Gadd153 in apoptosis. In this study, we sought to define in greater detail what role, if any, Gadd153/Bak plays in the regulation of cell death. We report that while Gadd153 expression alone does not trigger cell death, it does sensitize cells to killing by agents that stress the ER. Furthermore, we demonstrate that elevated Gadd153 expression up-regulates expression of Bak and the primary intracellular scavenger of ROS.

## Results

### Cd induces apoptosis of neuronal cells through ROS

**Figure 1:**
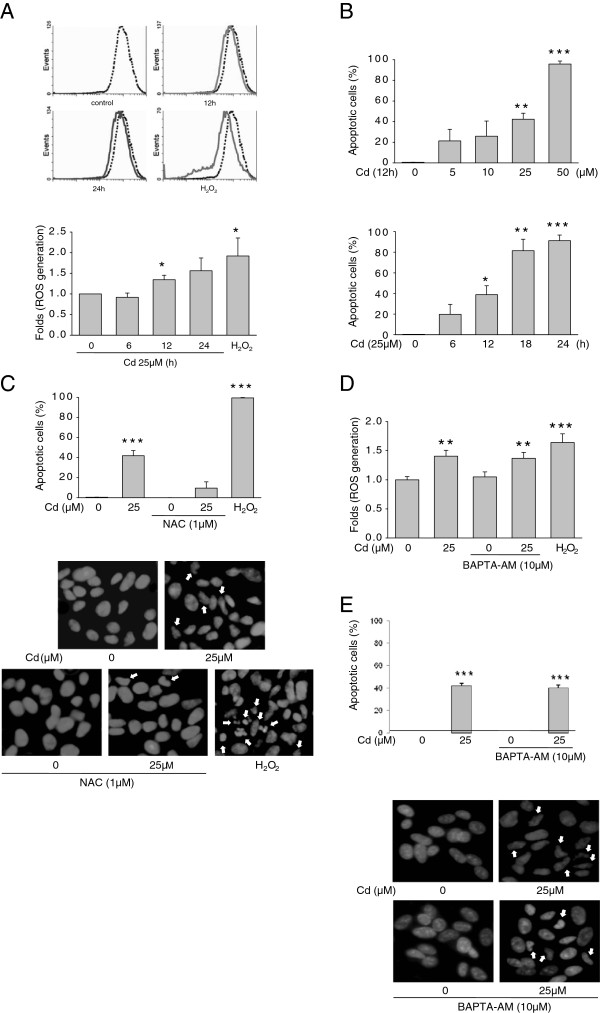
**CdCl**_**2 **_**induced ROS-generation and apoptosis in human neuroblastoma cell.** Representative flow cytometric analysis of intracellular ROS-generation in SH-SY5Y cells by 25 μM CdCl_2_ or 100 μM H_2_O_2_ treated for the indicated times (control;*dot line*, CdCl_2_ and H_2_O_2_; *solid line*) (*upper panel*). ROS-generation was measured using H_2_DCFDA as described in the materials and method and quantified (*lower panel*). (**A**). CdCl_2_-induced apoptosis measured by annexin V binding for various dose (*upper panel*) and time (*lower panel*) of CdCl_2_. Apoptotic cells were quantified. (**B**). SH-SY5Y cells were treated with or without a ROS scavenger, NAC (1 μM) for 1 hr and then incubated with CdCl_2_ (25 μM) or H_2_O_2_ for 12 hr. Apoptosis was measured by annexin V binding (*upper panel*) and DAPI staining (*lower panel*) (**C**). Annexin V binding apoptotic cells were quantified. Arrows indicated DAPI-positive cells shown fragmentation or nuclei condensation were observed in CdCl_2_ treated cells. SH-SY5Y cells were pretreated with a calcium chelater, BAPTA-AM (10 μM) for 30 min and then incubated with CdCl_2_ (25 μM, 12 hr). ROS-generation was quantified (**D**) and apoptosis was measured by DAPI staining (**E**). Data are presented as mean ± SEM, from at least three independent experiments. *p < *0*.*05*, **p < *0*.*01 or* ***p < *0*.*001 vs*. *control*.

Cd induces the production of ROS [11] and ER stress, which are importa nt triggers of the stress response in many cell types [12,13]. Previous report indicated involvement of ROS in Cd-induced renal cell injury [14]. To examine whether Cd trigger ROS in neuroblastoma cells, we investigated effects of Cd on the generation of endogenous ROS. Exposure of SH-SY5Y cells to Cd led to both 1.5 to 2 fold increase in intracellular ROS levels in a doses and time dependent manner (Figure 1A, B). To examine whether the generation of ROS is causative of Cd-induced apoptosis, cells were pretreated with antioxidant N-acetylcysteine(NAC) and subjected to fluorescent microscopy and DAPI staining (Figure 1C).

Increased cytoplasmic calcium [Ca^2+^ resulted either from calcium (Ca^2+^) influx from the extracellular environment or efflux from intracellular ER stores is associated with the initiation of apoptosis in diverse *in vivo* and *in vitro* systems [15]. To investigate the role of intracellular [Ca^2+^ in Cd-induced ROS generation, cells were pretreated for 12 hr with the Ca^2+^ chelator BAPTA-AM (10 μM) before treatment with 25 μM Cd treatment, Cd-induced ROS level is not changed (Figure 1D). In addition, BAPTA-AM is not effective in Cd-induced apop-tosis (Figure 1E). This indicates that ROS generation is not consistent with intracellular [Ca^2+^.

### Cd increases GADD153 and Bak expression via ROS

**Figure 2:**
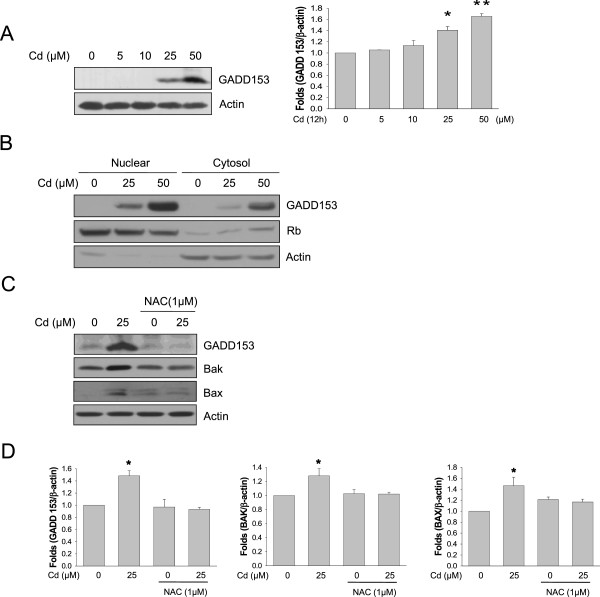
**CdCl**_**2 **_**increased GADD 153 and Bak expression through ROS-generation.** Level of GADD 153 was measured after CdCl_2_ (12 hr) for indicated dose-dependent concentration (**A**). Nuclear/Cytosol fractionation is indicated GADD153 nuclear translocation by CdCl_2_ (**B**). SH-SY5Y cells were treated with CdCl_2_ (25 μM) for 12 hr following pretreatment with NAC (1 hr). Total cellular protein was extracted and separated by SDS-PAGE. Western blot analysis was performed using anti-GADD 153 or Bak or Bax. Representative western blot band for GADD153, Bak and Bax expression were shown (**C**). Western blot bands were quantified (**D**). Data are from at least three independent experiments. *p < 0.05 or **p < 0.01 vs. control.

Cd induced apoptosis of SH-SY5Y cell is mediated, at least in part, by ER stress [16,17]. Dose dependent experiment revealed that induction of GADD153 was observed from 25 uM to 50 uM Cd treatment (Figure 2A). The activation of GADD153 leads to translocation of this transcription factor from the cytosol to the nucleus. Therefore, we examined the level of GADD153 after Cd treatment in nuclear extract. As shown Figure 2B, the level of GADD153 in nuclear extract is increased by Cd. To investigate relationship between oxidative stress and ER stress, SH-SY5Y cells were treated with Cd in the absence or presence of NAC, and expression of endogenous ER stress marker GADD153 was examined. Western blot analysis revealed that induction of GADD153 by Cd is completely attenuated by NAC (Figure 2C).

The cDNA array screen also identified the pro-apoptotic BCL2 family protein BAK as a fenretinide-inducible gene in SH-SY5Y cells, and this is confirmed by immunofluorescence flow cytometry and western blot data [18]. Studies on other cell types have shows that BAK can induce the release of cytochrome c from mitochondria, independently of mitochondria permeability transition, in combination with BH3 domain-only members of the BL2 family [19]. Therefore, the induction of BAK may be an event downstream of GADD153 induction leading to cytochrome c release and subsequent apoptosis in Cd-treated neuroblastoma cells.

To exclude a possibility that GADD153 is not located downstream of Bak, SH-SY5Y cells were treated with Cd in the absence or presence of NAC and expression of endogenous Bak was examined. Western blot analysis revealed that induction of Bak by Cd was partially attenuated by NAC (Figure 2C). Exposure of SH-SY5Y cells to Cd led to increase in intracellular Bax levels in a doses dependent manner (Figure 2C). These findings indicate that the GADD/Bak pathway plays a apoptosis role in Cd-induced cell death in SH-SY5Y cells.

### Knockdown of GADD153 downregulates Cd-induced apoptosis via Bak

**Figure 3:**
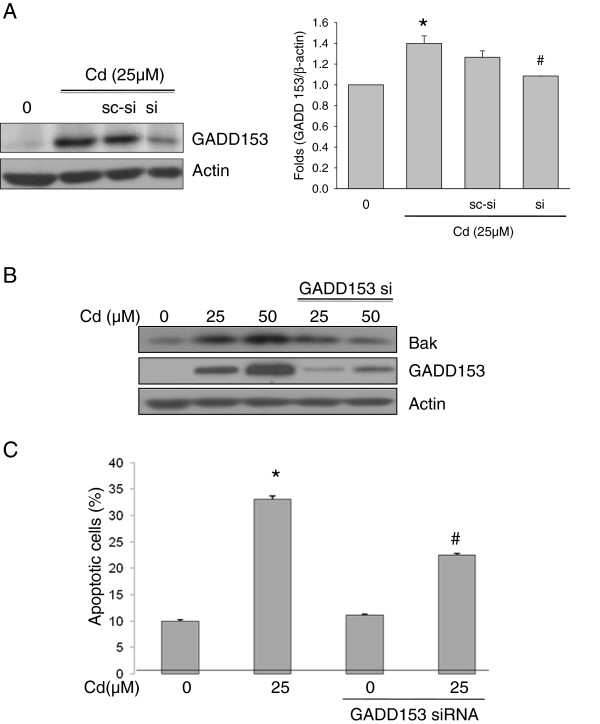
**CdCl**_**2 **_**increased Bak expression through GADD 153.**Transfected with scramble RNA or GADD-153 siRNA in SH-SY5Y cells were treated with CdCl_2_ (25 μM) for 12 hr and then total protein was extracted and separated by SDS-PAGE. Western blot analysis was performed using anti-GADD 153 antibodies (**A**). SH-SY5Y cells were treated with CdCl_2_ (indicate dose) for 12 hr following GADD-153 siRNA transfected. Bak and GADD153 expression level (**B**) and apoptotic cells (**C**) were measured. Data are from at least three independent experiments. *p < 0.05 vs. control,^*#*^*p < 0.05 vs. Cd treatment.*

Several studies have proposed that GADD153 activation results is apoptosis [20-22]. To investigate the role of GADD153 in Cd-induced apoptosis in SH-SY5Y cells, cells were transfected with small interfering RNA (siRNA) targeted against the GADD153 coding region and then treated with Cd (Figure 3A). As demonstrated in the literature, activation of Bak by Cd is decreased by knockdown of GADD153 (Figure 3B). Cd-induced apoptosis is decreased in GADD153 knockdown cells compared with control cell (Figure 3C).

### Chromatin immunoprecipitation reveals GADD153 binding to the Bak promoter

**Figure 4:**
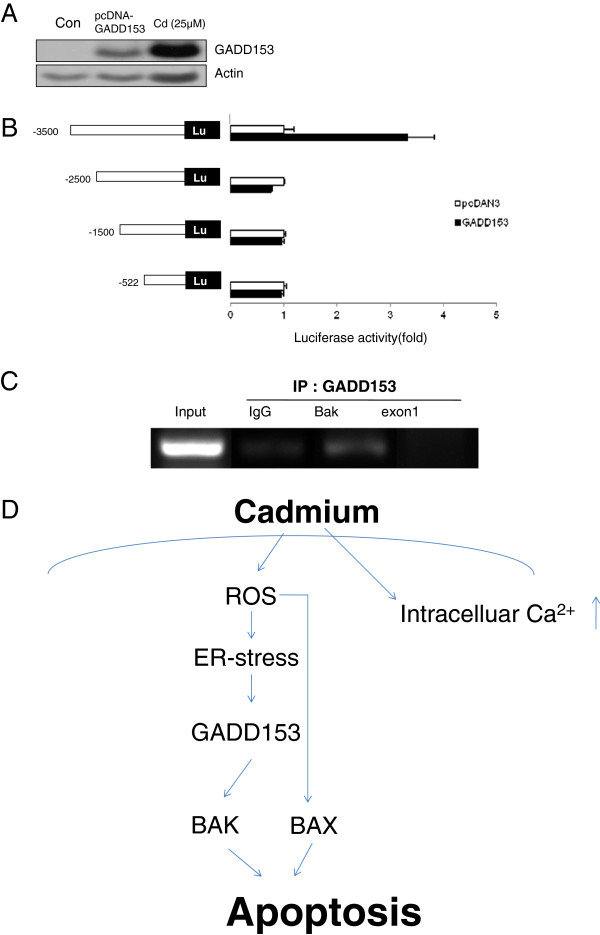
**GADD153 increased Bak expression level.** SH-SY5Y cells transfected with pcDNA3-GADD 153 or treated CdCl_2_ (25 μM, 12 hr). Total cellular protein was extracted and separated by SDS-PAGE. Western blot analysis was performed using anti-GADD 153 (**A**). Luciferase activitiy was measured 48 hr after co-transfected pcDNA3-GADD 153 and serial deleted Bak promoter region (**B**). SH-SY5Y cells were treated with 25 μM Cd for 12 h. Cells were cross-linking Histon-DNA complex by adding 37% formaldehyde for 30 min. Histon-DNA complex sheared between 200 and 1000 bp. ChIP products using specific antibodies directed against pre-immune IgG or GADD 153 were amplified by PCR. Input was sonicated chromatin prior to immunoprecipitation (**C**). Data are from at least three independent experiments. (**D**) Scheme of the proposed pathways mediating Cd-induced apoptosis.

To examine whether GADD153 increases Bak mRNA expression at the transcriptional level, we performed luciferase reporter gene assay. To localize the region of the Bak promoter responsible for GADD153 induction, the effects of serial deletion on Bak luciferase activity were analyzed in SH-SY5Y cells. Treatment with Cd and GADD153 Bak luciferase activity 3.2 ± 0.8 fold under the control of the −3,500 bp region versus the vector-transfected control, indicating that induction-fold decreased in the rest of untranslated region of Bak promoter (Figure 4A, B).

To study the mechanism of GADD153 to activate Cd- induced Bak transcription mediated transcription factors, the effect of GADD153 on the binding of C/EBP to the Bak promoters were analyzed ChIP assay in SH-SY5Y cells. As shown Figure 4C, basal constitutive GADD153 recruitment to the –3,398/–3,380 region of the Bak promoter was observed in SH-SY5Y cells.

## Discussion

ROS is implicated in heavy metal-induced apoptosis. The present study was performed to elucidate roles of ROS and downstream events induced-triggered apoptosis. Our results represented that Cd caused ER stress via generation of ROS and ER stress-induced apoptosis via activation of the GADD153/Bak pathway. The outline of our current hypothesis is summarized and illustrated in Figure 4D.

Previous reports indicated that Cd may induce apoptosis through altered activity of protein kinases, phosphatase and transcription factors, generation of ROS, induction of mitochondrial pathways or activation of caspases [23-25]. Mechanisms involved may be different from cell type to cell type, but among those, mitochondria have been regarded as the crucial target of Cd.

The relationship between oxidative stress and ER stress is not well understood. Haynes et al. reported that prolonged activation of UPR resulted in oxidative stress and consequent cellular death in *Saccharomyces cerevisiae*. Accumulation of ROS by UPR may be caused through two mechanisms; the oxidative folding machinery in the ER and the mitochondria dependent ROS generation [26]. However, our current results evidenced that oxidative stress was upstream, but not downstream of ER stress in cadmium-exposed cell. Consistent with our results, some reports also indicated that ER stress may be involved downstream of ROS [12].

GADD153 is a key control point apoptosis in apoptosis of various cell type, and since it is a transcription factor, GADD153 may be responsible for the induction of other genes required for Cd-induced apoptosis in neuroblastoma cells. The necessity for GADD153 in Cd-induced apoptosis has been confirmed by GADD153 si-RNA blocks the apoptotic response to Cd (Figure 3).

However, GADD153-siRNA doesn’t fully prevent Bak expression and apoptosis (Figure 3B). It is suggested that GADD153 activation may not be the sole mechanisms by which Cd cause cell death. Bax is another regulator in Cd-induced apoptosis (Figure 3C, 4C). Furthermore, level of Bcl-2 (downstream of GADD153-induced apoptosis) is not changed by Cd (data not shown). Our current finding that Cd-induced cell death raises a possibility that GADD153/Bak may also be involved not only in Cd-induced apoptosis but also in a variety of other biological responses.

The hypothesis that GADD153 is responsible for the induction of Bak was tested by protein-DNA interaction assay. We first hypothesized that putative C/EBP binding site may bind GADD153. The binding sites for the transcription factors seems to be localized in the Bak promoter region (−3,500 bp), because Bak luciferase was found to be increased in proportion to the extent of deletion in this region. The nuclear transcription factor GADD153 may form heterodimers with other C/EBP-family transcription factors and has been linked to a variety of cellular events including proliferation, differentiation and apoptosis [27]. The different members of C/EBP family can forms homodimers, heterodimers with another form of the C/EBPs and with other transcription factors that may or may not contain the leucine zipper domain [28].

We herein show that GADD153 is critical for Cd-induced apoptosis in neuroblastoma cells, Cd-induced Bak expression is mediated through a region between −3,398 and −3,380 of the Bak promoter. These results provide evidence for the first time that Cd-induced apoptosis of neuronal cells is mediated, at least in part, by ROS and that regulation via a distinct GADD153/Bak is implicated in the apoptotic process. Further investigation will be required to elucidate the spectrum of pathophysiological significance of the oxidative stress-ER stress axis.

## Conclusions

This study has examined the hypothesis that Cd induces neuronal cell death through ROS activated by GADD153. Our results are showing exposure of SH-SY5Y cells to Cd led to increase in intracellular ROS levels in a doses and time dependent manner. In addition, the generation of ROS and induction of GADD153 are causative of cadmium-induced apoptosis. GADD153 regulates Bak expression by its binding to promoter region (between −3,398 and −3,380). These results provide evidence for the first time that Cd-induced apoptosis of neuronal cells is mediated, at least in part, by ROS and that regulation via a distinct GADD153/Bak is implicated in the apoptotic process.

## Methods

### Materials

Dulbecco’s modified Eagle’s medium (DMEM), fetal bovine serum (FBS) and penicillin/ streptomysin were obtained from Gibco BRL (Gland Island, NY, USA). Cadmium chloride (Cd), NAC (N-acetyl-L-cysteine) were from Sigma (St. Louis, MO, USA). BAPTA-AM [1,2-bis(2-aminophenoxy) ethane-*N*,*N*,*N*’,*N*’-tetraacetic acid tetrakis (acetoxy methyl ester)] were obtained from Calbiochem-Novabiochem (San Diego, CA, USA). GADD153 antibodies were obtained from abcam (Cambridge, CB4 OFL, UK). Rb and Bax antibodies were obtained from Santa Cruz Biotechnology (Santa cruz, CA, USA). Luciferase assay kit was purchased from Promega (Madison, WI, USA). 2′,7′-dichlorodihydrofluorescein diacetate (2′,7′-dichlorofluorescein diacetate, H_2_DCFDA) were obtained from Molecular Probes (Eugene, OR, USA). pGL2 Bak promoter construct was kindly provided by Dr. Yong-Sung Juhnn(Seoul National University, Korea), pcDNA3-GADD153 expression construct was kindly provided by Dr. Dae-Ghon Kim(Chonbuk National University, Korea).

### Cell culture

Human neuroblastoma (SH-SY5Y) cells (ATCC, Rockville, MD, USA) were cultured in DMEM supplemented with 10% FBS, 100 Units/mL penicillin, and 100 μg/mL streptomycin. Cells were maintained in a 37°C /5% CO_2_ incubator.

### Measurement of reactive oxygen species

SH-SY5Y cells were treated with BAPTA-AM for 30 min and Cd treated for 11 hr 30 min. Cells were washed with phosphate-buffered saline (PBS), harvested using trypsin and washed with PBS. Resuspend cells in H2DCFDA (final concentration 20 μM) at 37°C incubator for 30 min. Cells were washed with PBS and then resuspend in PBS. ROS analyzed by flow cytometry (Beckman culter, Brea, CA, USA).

### Measurement of apoptosis by propidium iodide staining

SH-SY5Y cells were treated with Cd for 12 hour that they washed with PBS, harvested using trypsin. Cells were stained with propidium iodide (PI; 5 μg/mL) in PBS. They were analyzed by flow cytometry, and the extent of apoptosis was determined based on the sub-G1 population.

### Transfection and luciferase assay

SH-SY5Y cells were transfected with plasmid using Lipofectamine 2000 Reagent (Invitrogen, Camarillo, CA, USA) according to the manufacturer’s recommendation. Cells were incubated for 24 hr in 37°C 5% CO_2_ incubator. Luciferase activities were assayed using luciferase assay kit (Promega, Madison, WI, USA), according to the manufacturer’s instructions.

### Western blot analysis

Cells were lysed in RIPA buffer [50 mM Tris–HCl, pH 7.5, 1% NP-40, 0.5% sodium deoxycholic acid, 0.1% SDS, and protease inhibitors cocktail (sigma, St. Louis, MO, USA)] on ice for 20 min. Lysates were centrifuged at 12,000 × *g* for 15 min. Protein (60 μg) was separated by 10% SDS-PAGE, and the proteins were transferred onto a 0.45 μm PVDF membrane (Milipore, Bedford, MA, USA). After the membrane was blocked with 5% fat-free milk in TBST buffer (25 mM Tris–HCl, pH 7.4, 137 mM NaCl, 5 mM KCl, and 0.1% Tween 20) for 3 hr, it was incubated with the primary antibodies at 4°C overnight and secondary antibodies for 2 hr. All antibodies were used at a dilution of 1:1000. Membrane were developed using an enhanced SuperSignal West Femto Chemiluminescent Substrate (Thermo, Waltham, MA, USA).

### Nuclear/cytosol fractionation

Cells were treated with Cd for 12 hour and then washed two times with PBS, harvested using cell scraper. Cells were centrifuged at 300 × *g* for 4 min, resuspended buffer A (10 Mm HEPES, pH 7.5, 10 Mm KCl, 1 mM DTT, 1 mM PMSF, protease inhibitors cocktail) and incubated for 15 min on ice. Supernatant (cytosol) was collected by centrifuged at 4°C, 1500 × g for 5 min after 10 min incubation on ice with 10% NP-40. Pellet was washed two times with buffer A and then resuspended buffer B (buffer A + 0.4 M NaCl) for 30 min on ice. Supernatant (nuclear) was collected by centrifuged at 4°C, 15000 × g for 10 min.

### DAPI staining & annexin V assay

Cells were treated with an NAC for 1 h and then incubated with Cd for 12 h. After treatment with Cd, cells were washed with PBS, fixed for 30 min with 4% paraformaldehyde (PFA) prepared in PBS, followed by incubation with 4′,6-diamindine-2-phenilindole (DAPI; 10 μg/mL) for 30 min. They were analyzed by flow cytometry. Apoptotic cells with condensed or fragmented nuclei were visualized under a fluorescence microscope (Olympus Optical Co., Melville, NY, USA). Apoptotic cells were detected by annexin V assay kit (Molecular Probes, Inc., Eugene, OR, USA), according to the manufacturer’s instructions.

### ChIP assay

SH-SY5Y cells were routinely cultured and treated with 25 μM Cd for 12 h. Cells were fixed by adding 37% formaldehyde (final concentration 1%) directly to culture medium and incubate for 10 min. Cells were sonicated condition for 15 sec for18 time, 2 min-intervals, setting 35% output with micro-tip to keep sample on ice. Cells were detected by Chromatin immunoprecipitation assay kit (Millipore, Temecula, CA, USA), according to the manufacturer’s instructions. ChIP products using specific antibodies directed against pre-immune IgG (Santa cruze, Califonia, DA, USA) or GADD153. The purified DNA products were amplified target protein-DNA specific primer (Sense primer: 5′-AATCTAGTATTAGTATTCCCCA-3′, Anti-sense primer: 3′-ACCATTCTGGCTAACATGGTGA-5′) and non-specific primer (Sense primer: 5′-GTCTGCAT CCGGTGGCCACA-3′, Anti-sense primer: AACCCGGT CCTAGGGCCGTC).

### Statistical analysis

Data are expressed as the mean ± SD of experiments performed in triplicate and replicated at least three times. Data were evaluated with one-way analysis of variance (ANOVA) followed by Student’s *t*-test. Statistically significant differences are reported as *p < 0.05 or **p < 0.01. Data with values of p < 0.05 were generally accepted as statistically significant.

## Misc

Seungwoo Kim and Hyo-Soon Cheon contributed equally to this work.

## Competing interests

The authors declare no competing interests.

## Authors’ contributions

The work was carried out in collaboration between all authors. SW and HS carried out most of the experimental work, statistical analysis and wrote drafts of the manuscript. YS and SY carried out construction of Bak promoter vectors. YY supervised most of the experimental work and conceived of the study, participated in the finalized the manuscript. All authors have read and approved the final manuscript.
